# An Identical Twin Study on Human Achilles Tendon Adaptation: Regular Recreational Exercise at Comparatively Low Intensities Can Increase Tendon Stiffness

**DOI:** 10.3389/fphys.2021.777403

**Published:** 2022-01-05

**Authors:** Freddy Sichting, Nicolai C. Kram, Kirsten Legerlotz

**Affiliations:** ^1^Department of Human Locomotion, Chemnitz University of Technology, Chemnitz, Germany; ^2^Movement Biomechanics, Institute of Sport Sciences, Humboldt-Universität zu Berlin, Berlin, Germany

**Keywords:** Achilles tendon, connective tissue adaptation, genetic variation, exercise activities, aerial phase, sports, tendon stiffness, twin study

## Abstract

Achilles tendon adaptation is a key aspect of exercise performance and injury risk prevention. However, much debate exists about the adaptation of the Achilles tendon in response to exercise activities. Most published research is currently limited to elite athletes and selected exercise activities. Also, existing studies on tendon adaptation do not control for genetic variation. Our explorative cross-sectional study investigated the effects of regular recreational exercise activities on Achilles tendon mechanical properties in 40 identical twin pairs. Using a handheld oscillation device to determine Achilles tendon mechanical properties, we found that the Achilles tendon appears to adapt to regular recreational exercise at comparatively low intensities by increasing its stiffness. Active twins showed a 28% greater Achilles tendon stiffness than their inactive twin (*p* < 0.05). Further, our research extends existing ideas on sport-specific adaptation by showing that tendon stiffness seemed to respond more to exercise activities that included an aerial phase such as running and jumping. Interestingly, the comparison of twin pairs revealed a high variation of Achilles tendon stiffness (305.4–889.8 N/m), and tendon adaptation was only revealed when we controlled for genetic variance. Those results offer new insights into the impact of genetic variation on individual Achilles tendon stiffness, which should be addressed more closely in future studies.

## Introduction

In humans, the gastrocnemius muscle inserts together with the soleus muscle *via* a well-developed Achilles tendon onto the calcaneus (Swindler and Wood, 1973; Standring et al., 2016). Particularly the anatomical length of the human Achilles tendon is outstanding among extant great apes from a comparative perspective. In our closest relatives, the chimpanzees (*Pan troglodytes*) and gorillas (*Gorilla gorilla*), the gastrocnemius muscle inserts almost immediately onto the calcaneus, so that the Achilles tendon is barely visible (Swindler and Wood, 1973; Myatt et al., 2011). Several studies consider the well-developed Achilles tendon as an adaptation that fosters energy-efficient locomotion (Alexander, 1984, 1991), particularly during bipedal running (Bramble and Lieberman, 2004). Extensive research has shown that during running, the Achilles tendon acts like a spring element, which is stretched and loaded with strain energy during the initial phase of stance and recoils during the late phase of stance to support the foot push-off (Hof et al., 2002; Arampatzis et al., 2006; Lichtwark et al., 2007). While the evolutionary advantage of this adaptation seems generally accepted (Bramble and Lieberman, 2004), debate continues about structural adaptations of the Achilles tendon in response to exercise-related mechanical loading.

Generally, the Achilles tendon can adapt to external mechanical loading by increasing its stiffness, elastic modulus, and cross-sectional area (for review see Wiesinger et al., 2014; Bohm et al., 2015). The adaptation to external mechanical loading seems to depend on frequency, duration, and intensity. Data from several longitudinal training studies suggest that high muscle contraction intensities (i.e., 70–90% of maximum voluntary contraction) endured over several seconds are most effective in inducing structural changes within the tendon (for review see Bohm et al., 2015). It has been speculated that longer durations of tension during strength training exercises result in a more efficient transmission of the tendon strain *via* the extracellular matrix to the cytoskeleton of the tendon cells (Bohm et al., 2014). Consequently, the strain triggers cellular and molecular responses, such as the synthesis of collagen and matrix proteins, thereby affecting the Achilles tendon’s mechanical and morphological properties (Wang, 2006; Heinemeier and Kjaer, 2011; Galloway et al., 2013). The loading stimulus duration argument has recently been applied to explain why jumping results in less pronounced adaptive responses of Achilles tendon properties than an exercise protocol with longer loading duration (i.e., 3 s) (Bohm et al., 2014).

However, several cross-sectional studies on running and jumping report changes in the Achilles tendon’s cross-sectional area and/or stiffness, despite much shorter strain durations (i.e., 0.2–0.25 s). For example, Rosager et al. (2002) and Magnusson and Kjaer (2003) detected a significantly larger Achilles tendon cross-sectional area in trained runners than non-runners. Similarly, elite runners and volleyball players had larger Achilles tendon cross-sectional areas than elite kayak athletes, whose training includes less frequent mechanical stimulation of the lower extremities (Kongsgaard et al., 2005). In the same vein, Wiesinger et al. (2016) showed greater Achilles tendon cross-sectional areas in highly trained endurance runners and world-class ski jumpers compared to national-level elite water polo players and sedentary individuals. Notably, within the same study it was found that differences in the cross-sectional area were not consistently mirrored by differences in stiffness. Here, tendon stiffness was only higher in ski jumpers than in sedentary individuals but did not differ between runners and water polo players. In contrast, Karamanidis and Epro (2020) measured a significantly increased Achilles tendon stiffness in elite high and long jumpers compared to age-matched controls. Those results, although partly inconclusive, suggest that chronic exposure to repetitive loading by running and jumping can result in tendon adaptation.

An essential characteristic of running and jumping movements is a repetitive aerial phase, during which the athlete lifts both feet off the ground and then freely falls under gravity before touching the ground again (Zaciorskij, 2000). With touchdown, at the end of an aerial phase, the impact exposes the Achilles tendon to mechanical loading that can be magnitudes greater than during activities with no aerial phase, such as walking (Lichtwark et al., 2007; Lai et al., 2015; Kharazi et al., 2021). Particularly the pioneering work by Komi et al. (1987, 1992) and Fukashiro et al. (1995) revealed that Achilles tendon forces are highest in activities with an aerial phase. Also, the Achilles tendon undergoes considerable length changes (i.e., 5.6% for running and up to 8.2% for single-leg hopping) (Lichtwark and Wilson, 2005, 2006) within stretch-shortening cycles. While numerous research indicates that exercise activities with a regular aerial phase, respectively, exercise including stretch-shortening cycles, such as running and hopping, do not appear to induce immediate mechanical or morphological changes in the Achilles tendon (Obst et al., 2013), data on long-term tendon adaptation are rare. Regarding long-term effects, it is reasonable to speculate that individuals who regularly engage in exercise activities with an aerial phase (e.g., running, basketball, or tennis) show more pronounced Achilles tendon adaptations than individuals who engage in exercise activities without an aerial phase (e.g., cycling, inline skating, swimming).

Although there is evidence for enhanced adaptations in runners and jumpers, the generalizability of the existing research is problematic because those studies are often limited to elite athletes. Additionally, those studies do not control for genetic variation, even though there is evidence that the genome of elite athletes varies in some alleles compared to non-elite athletes (for review see Macarthur and North, 2005). Currently, it cannot be excluded that genetic variation also affects baseline Achilles tendon mechanical properties. One promising approach to overcome the challenge of genetic variation might be studying identical (monozygotic) twins. Identical twins derive from a single fertilized egg and inherit identical genetic material (Boomsma et al., 2002). There is a growing body of literature on identical twins that highlights the critical role played by genetic variation on strength and fitness outcomes (Marsh et al., 2020), bone metabolism (Smith et al., 2003), and exercise-induced muscle damage (Gulbin and Gaffney, 2002). However, no twin study has investigated the effect of exercise activities on Achilles tendon properties.

In light of the controversial literature on Achilles tendon adaptation and the potential impact of genetic variation, this study attempted to find evidence that long-time regular exercise activities, even at a recreational level, can trigger Achilles tendon adaptation. Another objective was to determine whether exercise activities with a regular aerial phase have greater effects on tendon adaptation. To this end, the experiment was designed as an explorative cross-sectional twin study with recreationally active or non-active identical twins. The experimental design allowed us to control for genetic variation. We measured Achilles tendon stiffness to evaluate tendon adaptation in response to regular exercise activities at a recreational level. Research shows that, in addition to tendon morphological properties such as the cross-sectional area, tendon mechanical stiffness increases in response to mechanical loading (Arampatzis et al., 2007; Bohm et al., 2014). Thus, changes in tendon stiffness are indicative of tendon adaptation. By comparing Achilles tendon stiffness among identical twin pairs, we aimed to test two hypotheses: (1) Regular recreational exercise activity leads to greater Achilles tendon stiffness compared to no regular exercise. (2) Exercise activities with an aerial phase lead to greater Achilles tendon stiffness than exercise activities without an aerial phase.

## Materials and Methods

### Participants and Experimental Design

Forty identical twin pairs (19 female and 21 male pairs) participated in this explorative cross-sectional study. All participants (mean age: 40 ± 18 years; body mass: 61 ± 18 kg; height: 162 ± 17 cm) were required to be healthy, with no injuries of the lower limbs within the last 6 months. Each participant gave written informed consent to participate in the study. The study was approved by the local ethics committee of the Faculty of Behavioral and Social Sciences and conducted according to the Declaration of Helsinki.

All participants answered a questionnaire, including questions about regular physical activity (“Do you participate in regular physical activity?”), kind of physical activity (“If yes, what kinds of physical activities do you participate in?”), weekly training load (“How many hours do you spend exercising each week?”), and, if they remembered, total years of training (“For how many years have you participated in this sport regularly?”). Participants were considered physically active if they followed a regular training regime of 60 min/week for at least 1 year, regardless of intensity. The different exercise activities were divided into sports with and without an aerial phase. Sports with an aerial phase had to be characterized by regular movements with both feet off the ground. Three investigators (KL, NCK, and FS) evaluated all sports independently and agreed with their decision on regular aerial phases. Sports without an aerial phase include bouldering, cycling, hiking, horse riding, nordic walking, pilates, resistance training (excluding plyometric training), speed skating, swimming, water gymnastics, weightlifting, and yoga. Sports with an aerial phase include indiaca, running, soccer, tennis, and trampoline.

**FIGURE 1:**
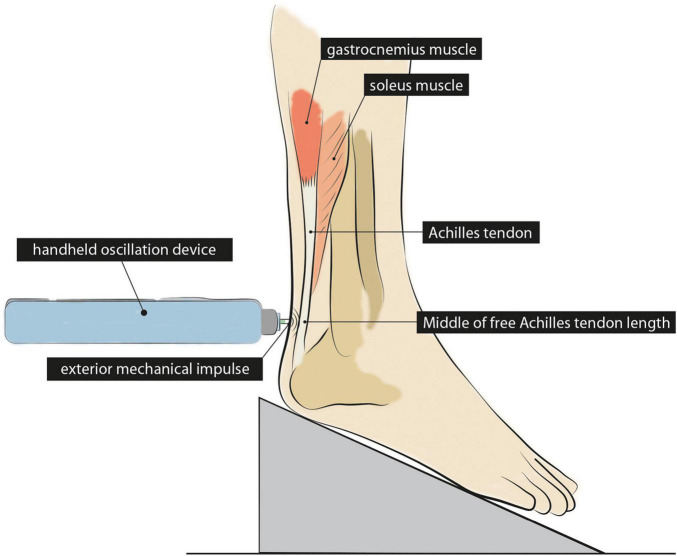
Illustration of the Achilles tendon stiffness measurement with the foot placed in a 20° dorsiflexed position. The stiffness of the Achilles tendon was measured in the middle of the free Achilles tendon length using a handheld oscillation device. The device applied a mechanical impulse to measure the oscillation response of the Achilles tendon as a surrogate measure of stiffness.

Once the questionnaire was answered, we determined and marked the middle of the free Achilles tendon in the twin pairs. Free Achilles tendon length is defined as the distance from the most distal insertion of the soleus muscle in the Achilles tendon to the distal insertion of the tendon at the calcaneus (Kongsgaard et al., 2005). According to Kongsgaard et al. (2005), the average free Achilles tendon length is about 10% of the lower leg length. Therefore, we measured the lower leg length in one of the identical twins in a standing position, as the distance from the palpated knee-joint gap (the gap between the femoral and fibular bone at the lateral side) to the floor. Then, we calculated the free Achilles tendon length (0.1 × lower leg length) and used that measure to draw a line from the palpated distal insertion of the tendon at the calcaneus toward the distal insertion of the soleus muscle (Figure 1). Next, we marked the middle of that line for the stiffness measurement. The same parameters were used to define the middle of the free Achilles tendon in the other twin, assuming anthropometric similarity in identical twins (Chatterjee et al., 1999). Within-pair variance in knee height of identical twins has been reported to be 0.24 cm (Chatterjee et al., 1999), suggesting that differences in Achilles tendon length are most likely equally small. Then, both twins had to sit on a chair with their knees bent at 90° and their feet positioned at a 20° dorsiflexion angle on a self-built construction (Figure 1). This position allowed us to measure the Achilles tendon stiffness in a relaxed state while applying a defined ankle angle (Davis et al., 1999; Orishimo et al., 2008; Hug et al., 2013; DeWall et al., 2014).

### Stiffness Measurement

To measure tendon stiffness, we used a handheld oscillation device (MyotonPRO^®^, Myoton AS, Tallin, Estonia). The device applies an external mechanical impulse to the surface of a tendon and, thereby, compresses the underlying tissue. An accelerometer then registers the response of the tendon in the form of a damped oscillation curve. Consequently, the amplitude and frequency of the sinusoidal curve are used as a surrogate measure of tendon stiffness (Schneider et al., 2015). The easily accessible and cost-effective device has already been used in previous studies to test its validity and reliability and to detect changes in Achilles tendon stiffness non-invasively (for review see Sichting and Kram, 2020). Regarding the quality of stiffness assessment, several studies have demonstrated that the use of the handheld device produces valid (Pruyn et al., 2016; Feng et al., 2018) and reliable results. Data consistently suggest good to excellent reliability for repeated measurements with intra-class correlations (ICC) of 0.87 (95% confidence interval: 0.61–0.96) (Ko et al., 2018), 0.83 (0.67–0.91) (Pruyn et al., 2016), and 0.90 (0.76–0.96) (Liu et al., 2018). Further, Schneebeli et al. (2020) reported a standard error of 10.8 N/m and a minimum detectable change of 30.0 N/m for intra-rater measurements of the relaxed Achilles tendon.

In our setup, we measured the Achilles tendon stiffness approximately in the middle of the free Achilles tendon. During each measurement, the device applied five consecutive short-term mechanical impulses (force: 0.4 N, impulse time: 15 ms), each separated by 1 s to allow for the vibrations to dissipate before the next impulse began. The mean of the five consecutive impulses was used to calculate stiffness. It should be noted that tendon stiffness derived from oscillation-based measurements differ quantitatively from stiffness values derived from ultrasound supported dynamometry.

### Statistics

We performed several analyses to test the hypothesis that regular recreational exercise activity leads to greater Achilles tendon stiffness than no regular exercise. We tested this hypothesis twice, (1) with and (2) without controlling for genetics. First, the unequal variance *t*-test of unrelated data (Welch’s *t*-test) (Ruxton, 2006) was applied to compare Achilles tendon stiffness in physically inactive and physically active twin pairs (both twins inactive vs. both twins physically active). This analysis aimed to test Achilles tendon adaptation to exercise activities without controlling for genetic predisposition. To control for age, we applied the Welch’s *t*-test to compare age in those twin pairs. Previous research has shown that age can affect Achilles tendon stiffness (Waugh et al., 2012; Delabastita et al., 2018). Another Welch’s *t*-test was applied to compare Achilles tendon stiffness in those twin pairs, in which twin A was regularly physically active while twin B was not (twin A vs. twin B). This analysis aimed to control for genetic predisposition. In addition to that, absolute differences in Achilles tendon stiffness were calculated for those twin pairs, in which both twins were physically inactive, and those twin pairs, in which twin A was regularly physically active, while twin B was not. The Welch’s *t*-test was applied to compare the two twin pairs. Finally, we plotted Achilles tendon stiffness of twin A against Achilles tendon stiffness of twin B for inactive twins pairs and those twin pairs, in which twin A was regularly physically active while twin B was not. Pearson correlation coefficients (r) were calculated to establish relationships between stiffness measurements of twin A and twin B. Correlations below 0.4 were qualitatively interpreted as weak, between 0.4 and 0.75 as moderate, and above 0.75 as strong (Fleiss, 1981).

For our second hypothesis, we predicted that individuals who engage in an exercise activity with an aerial phase show greater Achilles tendon stiffness than individuals who engage in an exercise activity without an aerial phase. To test this hypothesis, we compared mean stiffness differences in those twin pairs, in which both twins participated in a sport without an aerial phase against those twin pairs, in which at least one twin participated in a sport with an aerial phase using the Welch’s *t*-test. For all comparisons, we performed a power analysis to determine the effect size (Cohen’s d) of the results (≥ 0.8 = large; < 0.8– > 0.2 = medium; ≤ 0.2 = small) (Cohen, 2013). All statistical analyses were carried out using R Studio (R Foundation for Statistical Computing, Vienna, Austria). The level of significance was set at α = 0.05.

## Results

**FIGURE 2:**
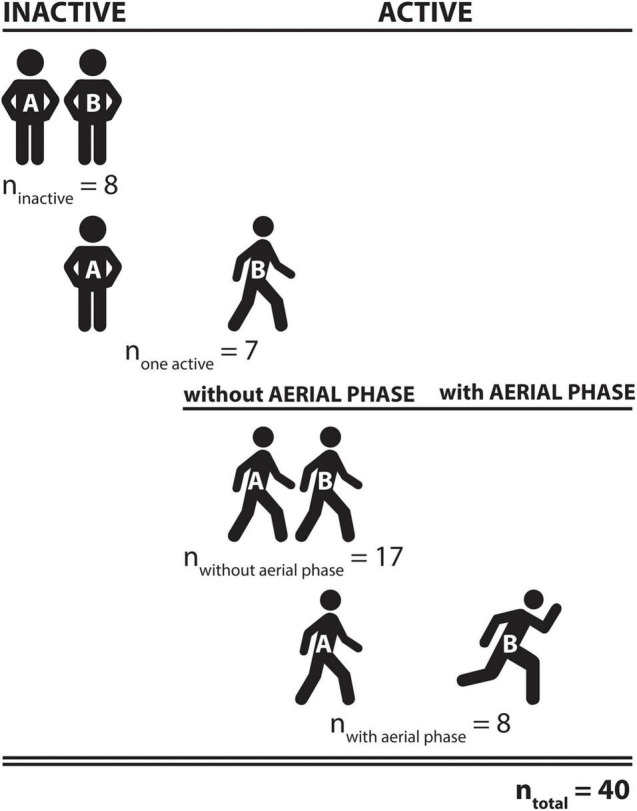
Summary of identical twin pairs who participated in the study. Among all 40 twin pairs, both twins were physically inactive in eight pairs, activity differed in seven pairs (twin A was regularly physically active, twin B was not), and both twins were regularly physically active in 25 pairs. The regular exercise activity in those 25 twin pairs included aerial phases in at least one of the twins activities in 8 pairs and no aerial phases in 17 pairs.

**FIGURE 3:**
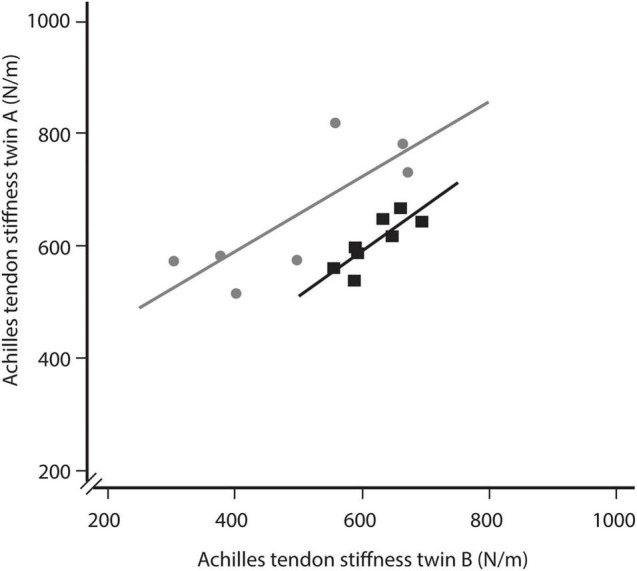
Comparison of Achilles tendon stiffness between identical twins, in which either both twins were inactive (black squares) or one twin was active while the other was not (gray dots). For the latter comparison twin A represents the active twin and twin B represents the inactive twin. The offset between the two regression lines indicates a greater Achilles tendon stiffness in the active twins.

Across all twin pairs (Figure 2), 17 different sports were performed for an average weekly duration of 4.0 ± 3.5 h. Thirty-eight participants were able to remember when they began their respective sport. Those participants performed their sport regularly for 16 ± 15 years (range: 2–45 years). Achilles tendon stiffness measures showed large variation between monozygotic twin pairs, ranging from 305.4 to 694.4 N/m in inactive twins, whereas in active twins measures ranged from 306.2 to 889.8 N/m (Figure 3).

**FIGURE 4:**
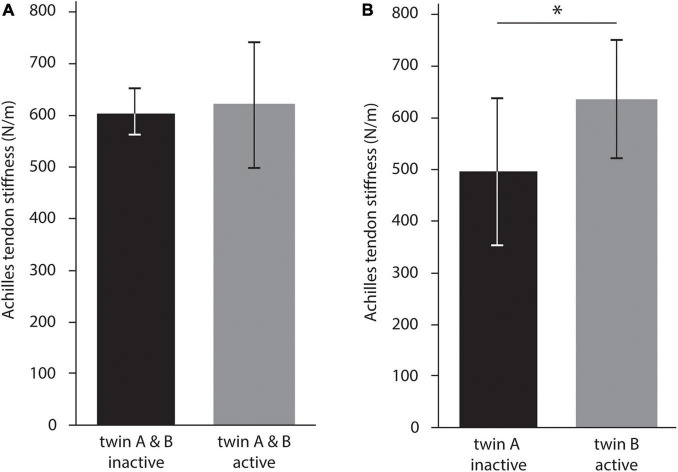
Analysis of the impact of regular exercise activity on Achilles tendon stiffness. **(A)** Compares inactive and active twin pairs. In contrast, **(B)** compares identical twin pairs where twin A is inactive and twin B is active. The latter comparison allowed to control for genetic predisposition and showed a significant difference, indicated by the asterisk (*p < 0.05).

Further comparison between physically active (both twins active) and inactive twin pairs (both twins inactive) did not reveal any significant differences (n_*active*_ = 25: 619.1 ± 121.5 N/m vs. n_*inactive*_ = 8: 605.0 ± 46.1 N/m) (Figure 4A). There was also no significant difference in age between those twin pairs (active: 41 ± 20 years vs. inactive: 36 ± 19 years). However, for twin pairs, in which only one twin was regularly physically active (n_*one active*_ = 7), Achilles tendon stiffness was significantly higher in the active (636.0 ± 115.5 N/m) compared to the inactive twin (496.8 ± 142.7 N/m), on average by 28.0% (*p* < 0.01) (Figure 4B). Cohen’s d was 0.59, indicating a moderate effect. The correlation analysis revealed a strong correlation between twin A and twin B, both for inactive twin pairs (*r* = 0.83) and those twin pairs, in which twin A was regularly physically active while twin B was not (*r* = 0.79) (Figure 3). Notably, the was a clear offset between the regression lines, representing inactive twin pairs and those twin pairs, in which twin A was regularly physically active while twin B was not. The offset might reflect the greater Achilles tendon stiffness in active twins compared to their inactive siblings.

**FIGURE 5:**
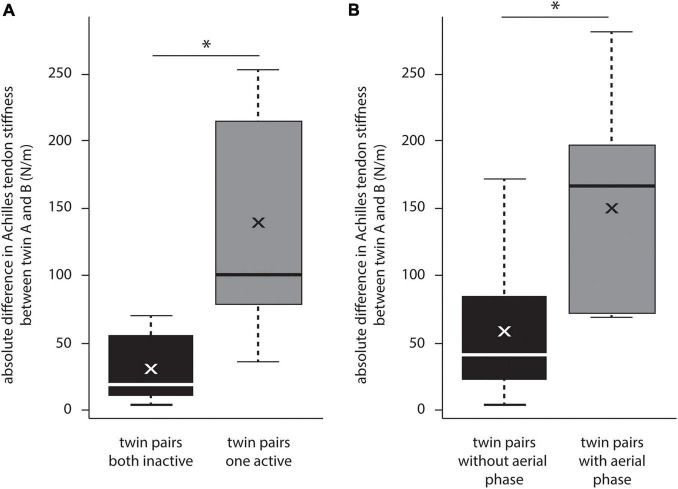
Comparison of Achilles tendon stiffness within identical twin pairs, expressed as absolute difference in Achilles tendon stiffness. **(A)** Shows the significant impact of regular exercise activities. **(B)** Shows the significant impact of an aerial phase on differences in Achilles tendon stiffness. Significant differences (*p* < 0.05) are indicated by an asterisk.

In terms of differences in Achilles tendon stiffness between identical twins, the mean absolute stiffness difference in inactive twin pairs was significantly smaller than in pairs with one active and one inactive twin (n_*inactive*_ = 8: 30.5 ± 26.1 N/m vs. n_*one active*_ = 7: 139.2 ± 86.6 N/m, *p* = 0.02) (Figure 5A). Cohen’s d was 0.84, indicating a large effect. Further, the mean absolute difference in Achilles tendon stiffness between identical twins who both performed a sport without an aerial phase was significantly smaller than between twins, in which at least one twin performed in a sport with an aerial phase (n_*no aerial phase*_ = 17: 57.9 ± 47.7 N/m vs. n_*aerial phase*_ = 8: 160.6 ± 74.6 N/m, *p* < 0.01) (Figure 5B). Cohen’s d was 0.96, indicating a large effect.

## Discussion

The present study was primarily designed to determine the effect of regular recreational exercise activity on Achilles tendon stiffness. To control for genetic variation, this study was conducted as an identical twin study. We identified a large variability in tendon stiffness among twin pairs, which seems at least partially genetically predetermined. When controlling for genetic preposition, the results of our study indicate that Achilles tendon stiffness was greatest in those individuals who regularly engaged in recreational exercise activities. Moreover, we found differences in Achilles tendon stiffness between individuals who engaged in an exercise activity with an aerial phase and those without an aerial phase. These findings add novel insights to the current understanding of tendon adaptation.

Our comparison of identical twin pairs indicates that Achilles tendon stiffness appears to be at least partially genetically predetermined. The correlation analysis of inactive twin pairs and twin pairs with one active twin revealed a strong linear relationship between identical twin pairs in Achilles tendon stiffness. Further, we found a considerable variation in tendon stiffness between twin pairs, ranging from nearly 300 N/m to a threefold of that tendon stiffness. Certainly, this variability between twin pairs would be expected if they were doing different levels of activity. However, against this prediction, we found a considerable overlap in tendon stiffness in inactive and active twin pairs (305.4–694.4 N/m vs. 306.2–889.8 N/m, respectively). This observation may support the hypothesis that Achilles tendon stiffness is partially genetically predetermined. Further, it lets us speculate about interindividual variation in Achilles tendon adaptation in response to mechanical loading. In this regard, data from genetic analyses indicate a significant interindividual variation in collagen fibril assembly and vulnerability to develop symptoms of chronic Achilles tendinopathy (Mokone et al., 2006; Hay et al., 2013). Related research by Passini et al. (2021) further shows that genetic mutations in the ion channel *PIEZO1*, which regulates the mechanosensitive function of tenocytes, can affect tendon properties. Similar results of a potential genetic predisposition have also been found for bone properties (Smith et al., 2003) and muscle strength (Marsh et al., 2020). While considerably more work needs to be done to understand the genetic influence on Achilles tendon stiffness and adaptation, our findings suggest that genetic predisposition should be considered when investigating tendon adaptation to exercise. The need for such a consideration of genetics becomes also apparent in our analyses of the impact of regular exercise activities on Achilles tendon stiffness. When we did not control for genetics by comparing physically inactive and active twin pairs (both twins inactive vs. both twins physically active), tendon stiffness was slightly but not significantly increased in active twin pairs (increased by 2.3%). In contrast, when we did control for genetics by comparing twin pairs, in which twin A was regularly physically active while twin B was not, tendon stiffness was significantly increased by 28.0% in the active twin. These findings on the role of genetics might help explain the controversial results on the effect of exercise activities in previously published cross-sectional studies.

Another important factor to consider when investigating tendon adaptation to exercise appears to be the presence of an aerial phase during movement. Our findings suggest that sports that include an aerial phase affect Achilles tendon stiffness more than sports without an aerial phase. Achilles tendon differences were greatest among twin pairs in which at least one twin participated in a sport with an aerial phase. In contrast, differences in Achilles tendon stiffness were relatively small when both twins participated in a sport with no aerial phase. This finding is consistent with data obtained by Wiesinger et al. (2016), who found that Achilles tendon stiffness in elite athletes with a regular aerial phase (ski jumpers) was considerably higher than in athletes without an aerial phase (water polo athletes). A potential influencing factor may be the stretch-shortening cycle of the Achilles tendon during exercise activities with an aerial phase. Previous research indicates that the stretch-shortening cycle in exercise activities with an aerial phase can be characterized by considerable stretching of the Achilles tendon during landing to store energy. Most of this energy is rapidly released during shortening to contribute to the power generated by the muscle-tendon unit during the propulsive phase (Maganaris and Paul, 2002; Ishikawa et al., 2005). Although there is evidence that those regular stretching stimuli have no immediate effect on Achilles tendon stiffness (Obst et al., 2013), there is much less information about long-term effects. However, research on long-term bone tissue adaptation might support the idea that exercise activities with an aerial phase have the greatest effect on Achilles tendon adaptation. A considerable body of literature shows that bone adaptation is greatest in high-impact sports, such as gymnastics, running, soccer, or rugby (e.g., Nordström et al., 1998; Daly et al., 1999; Lima et al., 2001; Morel et al., 2001; Scerpella et al., 2003; Yung et al., 2005; Maïmoun et al., 2013). While the underlying mechanism for the differences in bone adaptation is still part of an ongoing debate, it has been speculated that those high impacts are followed by bone deformation induced by high muscle forces pulling on the bone (Snow-Harter et al., 1990; Madsen et al., 1993; Nordström et al., 1998), which may result in larger adaptation. Large muscle forces pulling on the tendon during landing and propulsion may also help explain the observed differences in Achilles tendon stiffness in individuals who engaged in a sport with and without an aerial phase. However, our results also show large variation in Achilles tendon stiffness between twins being active in the same category of exercise. This variation might be explained by considerable differences in impact forces, acting muscle forces, and impact frequencies. For example, previous research has shown reasonable differences in Achilles tendon strain magnitude between participants who are forefoot or rearfoot strikers (Rice and Patel, 2017).

Interestingly, our findings on the impact of an aerial phase on Achilles tendon adaptation contrasts with an argument by Kharazi et al. (2021), who recently investigated Achilles tendon strain during walking and running. Their results let them speculate that submaximal running at up to 3.5 m/s does not provide sufficient tendon loading magnitude for triggering improvements of the Achilles tendon stiffness (Kharazi et al., 2021). This argument builds upon former studies that found no difference in Achilles tendon stiffness between runners and untrained individuals (Karamanidis and Arampatzis, 2006; Arampatzis et al., 2007). However, none of these studies controlled for genetic variation. Likely, those previous results were masked by a large natural variation in Achilles tendon properties. Our study allowed us to control for genetics. Particularly the comparison within inactive twin pairs supports the argument for a genetic predisposition. Here, we found a strong correlation and high similarity between twin A and twin B, with significantly smaller mean differences in Achilles tendon stiffness (30.5 ± 26.1 N/m) compared to twin pairs with one active and one inactive twin (139.2 ± 86.6 N/m). Also, there was a large variation between those inactive twin pairs, ranging from nearly 300 to almost 700 N/m. Most likely, the close similarity in Achilles tendon stiffness between twin A and twin B and the considerable variation between twin pairs can be explained genetically. That said, additional data from dizygotic twins could provide more definitive evidence for heritability in Achilles tendon stiffness (Boomsma et al., 2002).

Another finding that adds to the existing literature on exercise-related Achilles tendon adaptation refers to the activity level and response to habitual exercise. Currently, most research is limited to elite athletes (e.g., Rosager et al., 2002; Magnusson and Kjaer, 2003; Kongsgaard et al., 2005; Wiesinger et al., 2016; Karamanidis and Epro, 2020). In our study, however, all participants followed their exercise activity at a recreational level for an average weekly duration of 4.0 ± 3.5 h. Still, we found differences in Achilles tendon stiffness between active and non-active twins which could be attributed to habitual exercise. In contrast to this finding, Hansen et al. (2003) found no changes in Achilles tendon properties (i.e., cross-sectional area and stiffness) in previously untrained individuals after 9 months of habitual running (about 78 sessions and 43 h). Possibly, 9 months of habitual exercise were still not long enough to achieve measurable tendon adaptation, and tendons may need more time to adapt due to the generally low tissue turnover rate in tendons (Heinemeier et al., 2013). In our study, participants were considered active if they performed their sport regularly for at least 1 year. However, on average, they performed their sport regularly for more than 15 years (range: 2–45 years), which is a very long duration of regular mechanical stimulation of the tendon. The long exposure to regular activity raises the possibility that the Achilles tendon had sufficient time to adapt slowly to habitual exercise activities even at a recreational activity level, with structural adaptations accumulating and eventually becoming detectable. Thus, the investigation duration might be another factor besides genetic predisposition that can help to explain the controversial results on changes in Achilles tendon stiffness. Further research is required to fully understand the factors that trigger Achilles tendon adaptation in response to habitual exercise activities.

To build further confidence in our results more twin pairs with different athletic profiles (active vs. non-active, or aerial phase vs. no aerial phase) would help. Our study shows that most identical twins share a very similar lifestyle, including similar exercise activities. However, we did not collect data on exercise activities during childhood and adolescence. It is reasonable to speculate that exercise activities during childhood and adolescence might have affected tendon properties in adult individuals. Research on the life-long turnover of human tendon tissue indicates that the tendon core is formed during height growth and is essentially not renewed after that (Heinemeier et al., 2013). Thus, exercise activities during childhood and adolescence might be another factor that impacts Achilles tendon properties in adults. It should further be noted that our interpretation of the role of an aerial phase is currently limited to a comparison of twin pairs in which at least one twin performed in a sport with an aerial phase. A comparison of twin pairs in which one had performed an aerial phase sport while the other did not would have been a more valid analysis. Unfortunately, our sample of twin pairs included only two pairs who fulfilled that criterion. For that reason, we decided to run the analysis as presented. Additional caution should also be taken when interpreting the classification approach of the different sports. Here, we used a qualitative approach to classify the various exercise activities. Although this approach allows a relatively simple classification, it does not control or account for the variation in tendon load history. Future studies should aim to quantify impact forces and frequencies, as well as Achilles tendon strain.

Being limited to the handheld oscillation device for stiffness measurements, this study does not provide information about morphological adaptation, such as the tendons’ cross-sectional area or other relevant material properties, such as the elastic modulus. Those properties should be assessed to develop a more comprehensive understanding of tendon adaptation. That said, a study similar to this one could be carried out using ultrasound and dynamometry. Also, ultrasound would be a more precise method to determine free Achilles tendon length. Previous research indicates considerable variation in the length of the free Achilles tendon length (Drakonaki et al., 2021), but also between runners and non-runners (Devaprakash et al., 2020). Although we followed a standardized protocol to determine the middle of the free Achilles tendon, caution must be applied, as our approach does not guarantee that the stiffness measurements were acquired within the free portion of the tendon. Ultrasound could also help improving the definition of anatomical landmarks, like muscle-tendon or tendon-bone insertions. Advanced ultrasound technologies, like shear wave elastography, might be a consequent next step to measure stiffness properties along the complete free Achilles tendon, instead of using one measurement point as done in this study.

Notwithstanding these limitations, our study provides first evidence that the individual Achilles tendon stiffness is partially determined by genetic variation. In addition, the study has also indicates that exercise activities at a recreational level can trigger Achilles tendon adaptation if stimulated regularly for years. Our classification of exercise activities revealed that activities with a regular aerial phase seem to have the greatest impact on Achilles tendon adaptation. This finding extends an existing idea on sport-specific tendon adaptation presented by Wiesinger et al. (2016), which is currently limited to three sports activities (ski jumping, running, water polo) at an elite level. Our more general finding might also have implications for Achilles tendinopathy, an overuse injury of the Achilles tendon. When reviewing the literature on sports-related Achilles tendinopathy, the highest prevalence is reported for sports with a regular aerial phase, such as running, soccer, basketball, or rugby (Sobhani et al., 2013). Thus, it seems likely that regular exercise activities with an aerial phase can increase the risk of an overuse injury of the Achilles tendon. Consequently, individuals who participate in an exercise activity with an aerial phase should slowly increase their training intensity level to allow the Achilles tendon sufficient time to adapt to the high impact loading. Further, when performing a sport with an aerial phase, it might be helpful to include specific strength training sessions, as proposed recently by Radovanović et al. (2021). Such strategies could help to improve Achilles tendon properties and to prevent overuse injuries. That said, more research is needed to understand the impact of different exercise activities on Achilles tendon adaptation and the associated risk of injuries.

## Data Availability Statement

The raw data supporting the conclusions of this article will be made available by the authors, without undue reservation.

## Ethics Statement

The studies involving human participants were reviewed and approved by the Ethics committee of the Faculty of Behavioral and Social Sciences, Chemnitz University of Technology. The patients/participants provided their written informed consent to participate in this study.

## Author Contributions

FS and KL conceived the experiment, interpreted the data, and drafted the manuscript. NK and KL performed the experiments and substantially contributed to data analysis. FS analyzed the data. NK made important intellectual contributions during revision. All authors approved the final version of the manuscript and agreed to be accountable for the content of the work.

## Conflict of Interest

The authors declare that the research was conducted in the absence of any commercial or financial relationships that could be construed as a potential conflict of interest.

## Publisher’s Note

All claims expressed in this article are solely those of the authors and do not necessarily represent those of their affiliated organizations, or those of the publisher, the editors and the reviewers. Any product that may be evaluated in this article, or claim that may be made by its manufacturer, is not guaranteed or endorsed by the publisher.
